# Machine learning for predicting emergency department visits in patients with type 2 diabetes: A real-world, multi-institutional study

**DOI:** 10.1371/journal.pone.0352342

**Published:** 2026-07-09

**Authors:** Sunyoung Kim, Hyunji Sang, Jaeyu Park, Selin Woo, Eun-Hee Cho, Chong Hwa Kim, Dae Jung Kim, Chang-Won Jeong, Tae Sun Park, You-Cheol Hwang, Hyunjung Lim, Zio Kim, Hyejin Kang, Dong Keon Yon, Sang Youl Rhee

**Affiliations:** 1 Department of Family Medicine, Kyung Hee University College of Medicine, Seoul, Korea; 2 Center for Digital Health, Medical Science Research Institute, Kyung Hee University College of Medicine, Seoul, Korea; 3 Department of Endocrinology and metabolism, Kyung Hee University College of Medicine, Seoul, Korea; 4 Department of Regulatory Science, Kyung Hee University, Seoul, Korea; 5 Department of Internal Medicine, Kangwon National University College of Medicine, Chuncheon, Republic of Korea; 6 Department of Internal Medicine, Sejong General Hospital, Bucheon, Korea; 7 Department of Endocrinology and Metabolism, Ajou University School of Medicine, Suwon, Korea; 8 Smart Business Team in Information Management Office, Wonkwang University Hospital, Iksan, Korea; 9 Division of Endocrinology and Metabolism, Department of Internal Medicine, Research Institute of Clinical Medicine of Jeonbuk National University and Jeonbuk National University Hospital, Jeonju, Korea; 10 Division of Endocrinology and Metabolism, Department of Internal Medicine, Kyung Hee University Hospital at Gangdong and Kyung Hee University School of Medicine, Seoul, Korea; 11 Department of Medical Nutrition, Graduate School of East-West Medical Science, Kyung Hee University, Yongin, Korea; 12 Department of Data Science, Evidnet, Seoul, Korea; 13 Department of Precision Medicine, Kyung Hee University College of Medicine, Seoul, Korea; 14 Department of Pediatrics, Kyung Hee University Medical Center, Kyung Hee University College of Medicine, Seoul, Korea; University of Diyala College of Medicine, IRAQ

## Abstract

**Background:**

Patients with type 2 diabetes mellitus (T2DM) prone to acute diabetic complications are at high risk for emergency department (ED) visits, which often precede hospitalization and mortality. Identifying these high-risk phenotypes before deterioration is critical for preventative care. We developed machine learning (ML) models using large-scale, real-world electronic medical records, including prescription data, to predict the possibility of ED visits in patients with T2DM and support proactive interventions in primary care settings.

**Methods:**

We analyzed the electronic health record data of five independent institutions, creating a comprehensive dataset of 220,720 patients. The data included dynamic clinical parameters such as vital signs, laboratory results, and prescription histories. The cohort was randomly split into a training set (*n* = 176,576) and a test set (*n* = 44,144). The primary outcome was the first ED visit. We developed multiple ML models using an automated ML framework and optimized them using hyperparameter tuning of the training set. Model performances were evaluated using the area under the receiver operating characteristic (AUROC) curve, and feature importance was analyzed using SHAP values to ensure interpretability.

**Results:**

Among the screened population, 49,770 (22.6%) experienced at least one ED visit, distributed proportionally across the training and test datasets. The CatBoost model demonstrated superior predictive performance, achieving an AUROC of 0.87 (95% CI, 0.862–0.871) on the test dataset. The model identified modifiable risk factors as key predictors; Diastolic blood pressure was the most significant variable, followed by serum creatinine and systolic blood pressure.

**Conclusions:**

This ML-based predictive model can accurately identify high-risk patients with T2DM who are likely to visit the ED based on readily available clinical variables. By enabling healthcare providers to shift from reactive treatment to proactive risk management, it has the potential to reduce the burden of ED visits due to acute complications in T2DM.

## Introduction

The global population of individuals diagnosed with diabetes reached 828 million in 2022, with the number of adult patients increasing from 198 million in 1990–630 million, and the prevalence doubling from 7% to 14% [1]. While diabetes is primarily managed in outpatient settings, patients with type 2 diabetes mellitus (T2DM) are at substantially elevated risk for acute deteriorations that necessitate emergency care [2]. The most common precipitants of emergency department (ED) visits in this population include severe hypoglycemia — frequently triggered by excessive insulin dosing, missed meals, or declining renal clearance of insulin — and hyperglycemic emergencies such as diabetic ketoacidosis and hyperosmolar hyperglycemic state, which arise when insulin deficiency, infection, or other intercurrent illness disrupts glucose regulation [3,4]. These complications are particularly hazardous as they can rapidly progress to altered mental status, electrolyte imbalances, lethal arrhythmias, and mortality if not promptly corrected [5]. The annual rate of ED visits among adults with diabetes and two or more comorbid chronic conditions increased to 541.4 visits per 1,000 individuals between 2020 and 2021 [6]. The economic burden is substantial, as the average cost of an ED visit for a patient with diabetes is estimated to be twice to thrice higher than that of a primary care visit [7,8], contributing significantly to the overall healthcare expenditures and impacting patients’ safety owing to the risk of adverse events during acute episodes.

Critically, many of these ED presentations are preventable. Early identification of patients with warning signs — such as worsening glycemic control, declining renal function, escalating comorbidity burden, or high-risk medication patterns — would allow clinicians to intervene proactively through medication adjustment, intensified outpatient monitoring, patient education, or timely specialist referral. However, primary care providers often lack systematic tools to identify, among a large patient panel, which individuals are at imminent risk for acute decompensation requiring emergency care.

Healthcare providers must identify high-risk patients and intervene early by managing modifiable risk factors to mitigate the frequency and impact of ED visits [9]. This can be achieved with the use of accessible and up-to-date variables that can accurately predict ED utilization. Traditional prediction models depended on stepwise logistic regression and other statistical approaches based on predefined clinical variables, possibly limiting their ability to capture the multifaceted and dynamic nature of patient risk [10]. In contrast, machine learning (ML) models use nonparametric algorithms to integrate complex, high-dimensional data, thereby enhancing predictive accuracy and identifying novel risk factors [11]. ML-based approaches can outperform conventional models by reducing errors, lowering computation time and costs, and improving the quality of healthcare delivery [12]. However, most previous research has been limited to single-institution datasets or has not directly compared the performance of ML models to traditional statistical methods in predicting ED visits among patients with type 2 diabetes mellitus (T2DM).

Electronic medical records (EMR) provide a comprehensive repository of patient information, including demographics, clinical histories, prescriptions, laboratory results, and regularly updated clinical measurements, allowing for robust and timely analyses [13]. The EMR data integration into predictive modeling enables the development of personalized and adaptive interventions, potentially reducing unnecessary ED visits, lowering healthcare costs, and improving patient outcomes.

This study addresses the limitations of previous research [10–12] by developing and internally validating a ML-based prediction model for ED visits among patients with T2DM, utilizing large-scale, multi-institutional EMR data. Specifically, we (1) compared the predictive performance of ML models with traditional statistical approaches and (2) identified the key risk factors associated with ED utilization in this population.

## Methods

### Study population and data collection

This retrospective study used integrated data from five medical institutions enrolled in a multicenter observational study. Hospital-based data were collected from January 1, 2008, to December 31, 2022. Eligible participants were selected from patients with T2DM, and those with type 1 diabetes were excluded. A total of 220,720 patients were included in the study. The number of patients from each hospital was as follows: Kyung Hee Medical Center (KHMC, n = 64,436), Ajou University Medical Center (AUMC, n = 68,932), Wonkwang University Hospital (WKUH, n = 31,263), Bucheon Sejong Hospital (SJMC, n = 38,195), and Kangwon National University Hospital (KNUH, n = 17,894).

All participating institutions had previously transformed their source EMR data into the Observational Medical Outcomes Partnership Common Data Model (OMOP-CDM), which provides a uniform relational schema together with standardized vocabularies (e.g., SNOMED CT, RxNorm, and LOINC), thereby standardizing the structure of electronic health record (EHR) data across heterogeneous hospital systems. The analytic registry for this study was generated by extracting the required variables directly from each institution’s local CDM instance, ensuring that all data elements were structurally and semantically harmonized at the point of integration. Importantly, all patient records were de-identified and anonymized at the source institution prior to data transfer under this framework, such that the research team had no access to information that could identify individual participants at any stage of data collection or analysis.

### Prediction framework

The temporal framework of the prediction task was defined as follows. The index date for each patient was set as the date of the first recorded T2DM diagnosis in the EMR, with one record retained per patient. The look-back window for predictor ascertainment was defined as a one-year period centered on the index date (±365 days); all candidate variables — including demographics, comorbidities, medication exposure, vital signs, and laboratory test results — were extracted from this window. For variables with repeated measurements within the window, the median value was used as the feature, and the within-patient standard deviation was retained as an additional variability feature. The prediction horizon was defined as the occurrence of the first ED visit within the same one-year peri-diagnosis window. Accordingly, the model characterizes the clinical risk profile associated with ED visit occurrence over the one-year period surrounding the T2DM diagnosis date.

### Outcome definition and group classification

The primary outcome was defined as all-cause ED utilization rather than ED visits restricted to diabetes-specific causes (e.g., hypoglycemia, hyperglycemic crises, diabetic ketoacidosis).

Both the ED visit and non-ED visit groups were derived from the same unified multi-institutional T2DM registry using identical eligibility criteria; no separate recruitment or data collection procedure was employed for either group. An ED visit patient was defined as any eligible patient with at least one documented emergency department encounter in the EMR during the one-year peri-diagnosis window, with only the first ED encounter retained per patient. A non-ED visit patient was defined as an eligible patient with no documented ED encounter during the same window.

### Input variables

A total of 55 variables were included in the predictive model. Baseline patient demographics included age, sex, and medical history, including hypertension, dyslipidemia, macrovascular complications (ischemic heart disease, myocardial infarction, heart failure, atrial fibrillation, Parkinson’s disease, dementia, cerebrovascular disease, peripheral vascular disease, and lower limb amputation), microvascular complications (retinopathy, proliferative diabetic retinopathy, chronic kidney disease, end-stage renal disease, and neuropathy), and cancer. Medication history included types of antidiabetic agents (metformin, sulfonylurea, dipeptidyl peptidase-4 inhibitor, meglitinide, thiazolidinedione, α-glucosidase inhibitor, insulin, glucagon-like peptide-1 receptor agonist, and sodium-glucose co-transporter 2 inhibitor), antihypertensive drugs (angiotensin II receptor blocker, angiotensin-converting enzyme inhibitor, calcium channel blocker, diuretics, and beta-blockers), dyslipidemia drugs (statin, fibrate, and ezetimibe), and antiplatelet agents (aspirin, clopidogrel, cilostazol, and glycoprotein IIb/IIIa antagonist). Clinical parameters included systolic blood pressure, diastolic blood pressure, pulse rate, and body mass index (BMI) as continuous variables [14]. Blood tests included glucose, glycated hemoglobin (HbA1c), total cholesterol, triglyceride, high-density lipoprotein (HDL) cholesterol, low-density lipoprotein (LDL) cholesterol, serum creatinine, aspartate aminotransferase (AST), alanine aminotransferase (ALT), gamma-glutamyl transferase (GGT), and alkaline phosphatase (ALP) as continuous variables.

### Data preprocessing

**Fig 1:**
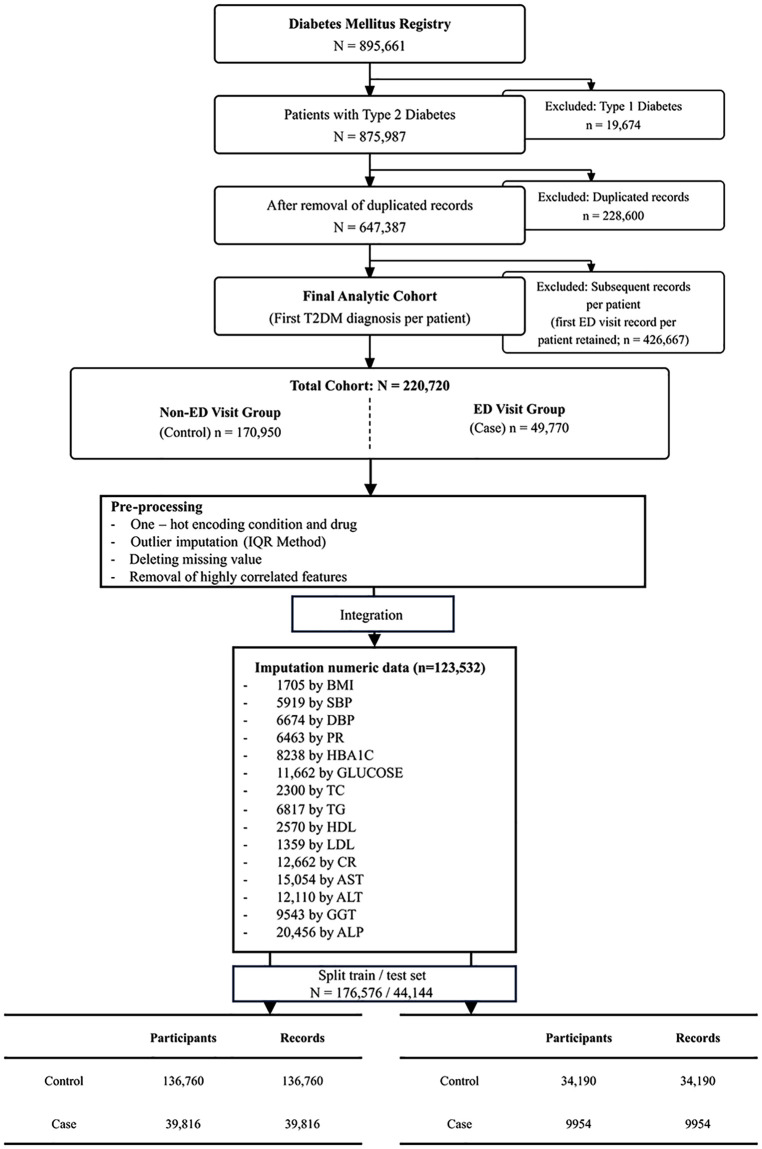
Study workflow. All available records of participants with and without a history of emergency visits were included. ALP, alkaline phosphatase; ALT, alanine transaminase; AST, aspartate aminotransferase; BMI, body mass index; CR, creatinine; DBP, diastolic blood pressure; GGT, gamma-glutamyl transferase; Glucose, plasma glucose; HDL, high-density lipoprotein cholesterol; IQR, interquartile range; HbA1c, glycated hemoglobin; LDL, low-density lipoprotein cholesterol; PR, pulse rate; SBP, systolic blood pressure; TC, total cholesterol; TG, triglyceride.

The Diabetes Mellitus Registry, comprising 895,661 patients, was refined using several preprocessing steps. First, the patients with type 1 diabetes were excluded. Secondly, duplicate patient records were excluded. We retained the first ED visit record per patient to avoid duplicate entries and ensure observational independence. Outliers were handled using the interquartile range (IQR), where values below the first quartile minus 1.5 times the IQR or above the third quartile plus 1.5 times the IQR were replaced with the corresponding boundary values. Finally, the missing values in the numerical variables were imputed using a multiple imputation method (iterative imputation). The missingness rates for all 15 continuous predictor variables are summarized in S1 Table. This approach was selected to mitigate the influence of extreme values while preserving all data entries for model training. Median values were used for the initial imputation. The imputed values were constrained within the original feature range to preserve the statistical characteristics of the data. Categorical variables had no missing values and were not imputed (Fig 1).

### Statistical analysis

Continuous variables are presented as means and standard deviations, whereas categorical variables are described using frequencies and percentages. Standardized mean differences (SMD) were calculated for all variables to assess the practical significance of group differences. An SMD > 0.2 indicated a meaningful difference between patients who visited the emergency department and those who did not.

### Model training and validation

The full cohort (n = 220,720) was partitioned once into a training set (80%, n = 176,576) and a hold-out test set (20%, n = 44,144) using outcome-stratified random sampling with a fixed random seed. The training set was used exclusively for all model development steps, including data preprocessing, feature selection, algorithm training, and hyperparameter tuning performed within the MLJAR AutoML framework; model selection during development relied on AutoML’s internal cross-validation on the training set. The hold-out test set was sequestered throughout model development and used only once, after the final model had been fixed, to provide an unbiased estimate of generalization performance (area under receiver operating characteristic [AUROC], area under the precision-recall curve [AUPRC], sensitivity, specificity, and related metrics) [15]. This procedure constitutes internal hold-out validation rather than external validation.

### Model development

We employed the MLJAR AutoML (automated machine learning) framework for automated model development [16]. AutoML streamlines the ML model development by automating complex tasks, such as data preprocessing, feature selection, model training, and hyperparameter optimization. This approach is particularly effective for achieving efficient modeling of large-scale datasets. The framework systematically explores different algorithms and optimizes their hyperparameters; tuned hyperparameters are summarized in S2 Table. The models primarily consist of decision-tree-based ensemble models, which combine ML with multiple models to achieve superior predictive performance and stability compared to individual models, such as Decision Tree, extreme gradient boosting (XGBoost), Random Forest, light gradient boosting machine (LightGBM), categorical boosting (CatBoost), and extremely randomized trees (ExtraTrees), as well as a deep learning method using neural networks [17,18].

### ML analysis

We employed MLJAR (version 0.10.3), an AutoML framework, to develop models for predicting new ED visits. MLJAR is a comprehensive AutoML tool designed specifically for tabular data that can handle the entire machine-learning pipeline from preprocessing to model evaluation [19]. As part of this automated pipeline, hyperparameter tuning was performed for each algorithm using a combination of random search, the Optuna framework [20], and hill-climbing strategies to identify optimal model configurations. Optuna is distinguished by its ability to explore high-dimensional parameter spaces in large-scale datasets effectively. Optimizing hyperparameters significantly influencing model performance, such as the learning rate, network depth, and activation functions, facilitates higher diagnostic accuracy.

**Fig 2:**
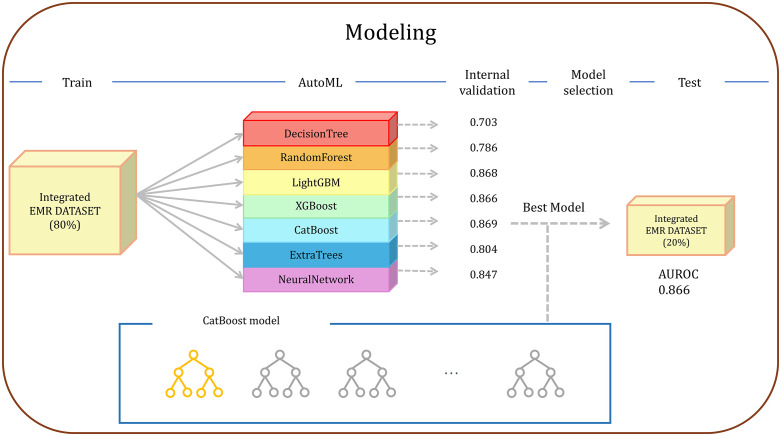
Model architecture. An integrated electronic medical record dataset from five institutions was used to develop a prediction model using an automated ML-based framework. The training dataset (80%) was used to train multiple ML algorithms, including Decision Tree, Random Forest, LightGBM, XGBoost, CatBoost, ExtraTrees, and neural networks. Among these, the CatBoost model demonstrated the best performance based on the hold-out validation (AUROC = 0.869) and was selected as the final model. The model was further evaluated using a test set (20%).

First, a baseline model was developed as a performance reference. Subsequently, each candidate algorithm underwent hyperparameter optimization within the AutoML framework. Next, the best-performing model was selected based on its AUROC in the internal validation to assess the generalizability of the model. The selected model’s performance was evaluated using several metrics, including AUROC, AUPRC, accuracy, sensitivity, specificity, positive predictive value (PPV), and negative predictive value (NPV), and was calculated using the optimal probability threshold identified by Youden’s Index [21]. The AutoML framework was used to develop seven models—Decision Tree, XGBoost, Random Forest, LightGBM, CatBoost, Extra Trees, and Neural Network—based on the training data (Fig 2).

Among the different ML models that underwent hold-out validation, the CatBoost model yielded the highest AUROC score and was selected to identify the most important features for predicting ED visits. The feature importance was extracted using the feature importance attributes of the CatBoost model. The top 20 features with the most significant impact on the model were selected and visualized using a bar graph to highlight their influence on model predictions.

### Performance metrics

To comprehensively characterize the predictive performance and clinical utility of the developed models, a range of discrimination, calibration, and clinical decision metrics were assessed. The AUROC curve is a robust measure of a model’s ability to discriminate between classes across all possible thresholds, because it considers both sensitivity and specificity, making it particularly valuable in scenarios with class imbalance. Although accuracy, which indicates the proportion of true results (both true positives and true negatives) among the total number of cases examined, is simple and intuitive, it can be misleading for unbalanced datasets, necessitating additional metrics. Sensitivity and specificity provide insights into the model’s ability to identify positive and negative cases, respectively. Sensitivity measures the proportion of true positives correctly identified by the model and reflects its ability to detect positive cases. In contrast, specificity measures the proportion of correctly identified true negatives, indicating the model’s capacity to avoid false alarms. These metrics can be combined to ensure a comprehensive and robust performance evaluation from multiple perspectives [22]. The AUPRC was additionally reported as a complementary discrimination metric, given its greater sensitivity to model performance on the minority positive class under class imbalance.

The classification threshold required to convert predicted probabilities into binary class labels was selected using Youden’s J statistic computed on the hold-out validation set [21], and this same threshold was applied for all subsequent calculations of accuracy, sensitivity, specificity, PPV, and NPV. The AUROC was reported as a threshold-independent measure of discrimination. Two-sided 95% confidence intervals (CIs) were calculated for each metric as follows: AUROC using DeLong’s nonparametric method [23], and all proportion-based metrics (accuracy, sensitivity, specificity, PPV, NPV) using the exact binomial (Clopper–Pearson) method.

In addition to discrimination metrics, calibration of the final CatBoost model was assessed to evaluate the agreement between predicted probabilities and observed event rates, as calibration is essential for clinical prediction models intended for individualized risk stratification [24,25]. Calibration was quantified using the Brier score — a composite measure of prediction accuracy incorporating both discrimination and calibration components [26] — as well as the calibration slope and calibration intercept, and was visualized using a calibration plot. Clinical utility of the model was evaluated using decision curve analysis (DCA), which calculates the net benefit of a prediction model across a range of threshold probabilities, comparing the model against the default strategies of treating all or treating none [27].

### Software and libraries

Data preprocessing, model development, and analyses were conducted using Python 3.10.1 (Python Software Foundation; Wilmington, DE, USA). The main libraries used in our study included Scikit-learn1.5.1, NumPy 1.26.4, and Pandas 2.2.2 for ML algorithms and data manipulation. Matplotlib 3.9.2 and Seaborn 0.13.2 were used for data visualization.

### Ethical approval

This study was approved by the Institutional Review Board of the University Hospital (approval no. KHSIRB-22–473 (EA)), which waived the requirement for informed consent because of the use of de-identified and anonymized data processed through a CDM framework for analysis. The data were accessed for research purposes on April 2, 2025. The authors had no access to information that could identify individual participants during or after data collection.

## Results

### Cohort baseline characteristics

Among patients in the original dataset, 220,720 patients, 49,770 (22.6%) visited the ED. Among these, 119,176 (54.0%) were male, with a mean age of 62.1 ± 13.8 years and a BMI of 25.1 ± 2.5 kg/m² (Table 1). Comparisons between the ED visit and non-ED visit groups identified several variables with a meaningful between-group difference (SMD > 0.2). Patients who visited the ED were older (64.6 ± 14.2 vs. 61.5 ± 13.6 years), had higher fasting glucose (159.7 ± 58.6 vs. 146.7 ± 43.9 mg/dL) and serum creatinine levels (1.0 ± 0.3 vs. 0.9 ± 0.2 mg/dL), and lower total cholesterol (155.9 ± 43.6 vs. 167.1 ± 34.4 mg/dL), HDL cholesterol (44.8 ± 10.4 vs. 47.5 ± 9.9 mg/dL), and LDL cholesterol (88.0 ± 33.5 vs. 94.8 ± 28.3 mg/dL). A markedly higher proportion of ED visitors used insulin (46.1% vs. 21.2%), and were more frequently prescribed diuretics (39.5% vs. 21.5%) and calcium channel blockers (47.8% vs. 32.2%). ED visitors also had a higher prevalence of hypertension (80.2% vs. 65.2%) and cerebrovascular disease (19.5% vs. 10.1%). These findings suggest that ED visits in patients with T2DM are associated with a more complex clinical profile characterized by poorer glycemic control, greater comorbidity burden, and heavier pharmacological burden. Site-level baseline characteristics for each of the five participating institutions are presented in S3 Table.

**Table 1:** Baseline characteristics of the study population by ED visit.

	Original dataset			
	Overall (220,720)	Non-ED visit (170,950)	ED visit (49,770)	SMD
Age, year, mean (SD)	62.2 (13.8)	61.5 (13.6)	64.6 (14.2)	0.223
Male, *n* (%)	119,176 (54.0)	91,267 (53.4)	27,909 (56.1)	0.054
BMI, kg/m^2^, mean (SD)	25.1 (2.5)	25.1 (2.4)	24.9 (2.5)	0.107
Systolic BP, mmHg, mean (SD)	126.3 (16.7)	126.6 (11.5)	125.1 (27.9)	0.071
Diastolic BP, mmHg, mean (SD)	74.8 (10.0)	75.0 (7.4)	74.1 (15.9)	0.077
Pulse rate, bpm, mean (SD)	78.8 (11.7)	78.3 (8.6)	80.3 (18.8)	0.135
Blood test, mean (SD)				
Glucose, mg/dL	149.7 (47.9)	146.7 (43.9)	159.7 (58.6)	0.252
Total cholesterol, mg/dL	164.6 (37.0)	167.1 (34.4)	155.9 (43.6)	0.283
HDL cholesterol, mg/dL	46.8 (10.0)	47.5 (9.9)	44.8 (10.4)	0.267
LDL cholesterol, mg/dL	93.2 (29.7)	94.8 (28.3)	88.0 (33.5)	0.218
Serum creatinine, mg/dL	0.9 (0.3)	0.9 (0.2)	1.0 (0.3)	0.289
ALP, U/L	87.8 (33.5)	88.0 (33.1)	87.4 (35.1)	0.016
AST, U/L	26.3 (9.3)	26.1 (8.7)	27.0 (11.2)	0.095
ALT, U/L	25.9 (13.1)	26.1 (12.6)	25.2 (14.9)	0.061
GGT, U/L	43.3 (26.3)	42.5 (24.8)	46.3 (30.6)	0.139
HbA1c, %	7.1 (1.1)	7.0 (1.1)	7.2 (1.2)	0.130
Triglyceride, mg/dL	146.7 (61.0)	147.5 (60.7)	144.0 (61.8)	0.058
Co-morbid conditions, *n* (%)				
Hypertension	151,314 (68.6)	111,381 (65.2)	39,933 (80.2)	0.343
Dyslipidemia	112,486 (51.0)	87,544 (51.2)	24,942 (50.1)	0.022
Macrovascular complications, *n* (%)				
Ischemic heart disease	38,314 (17.4)	29,658 (17.4)	8,656 (17.4)	0.001
Myocardial infarction	1,419 (0.6)	868 (0.5)	551 (1.1)	0.067
Heart failure	10,916 (5.0)	7,718 (4.5)	3,198 (6.4)	0.084
Atrial fibrillation	10,833 (4.9)	7,908 (4.6)	2,925 (5.9)	0.056
Parkinson’s disease	1,377 (0.6)	898 (0.5)	479 (1.0)	0.051
Dementia	2,314 (1.1)	1,716 (1.0)	598 (1.2)	0.019
Cerebrovascular disease	26,882 (12.2)	17,193 (10.1)	9,689 (19.5)	0.268
Peripheral vascular disease	483 (0.2)	291 (0.2)	192 (0.4)	0.041
Lower limb amputation	600 (0.3)	223 (0.1)	377 (0.8)	0.094
Microvascular complications, *n* (%)				
Retinopathy	9,649 (4.4)	7,511 (4.4)	2,138 (4.3)	0.005
Proliferative diabetic retinopathy	1,810 (0.8)	1,461 (0.9)	349 (0.7)	0.017
Chronic kidney disease	15,687 (7.1)	10,190 (6.0)	5,497 (11.0)	0.183
ESRD	846 (0.4)	423 (0.3)	423 (0.9)	0.082
Diabetic neuropathy	21,431 (9.7)	17,532 (10.3)	3,899 (7.8)	0.085
Cancer, *n* (%)	8,947 (4.1)	7,115 (4.2)	1,832 (3.7)	0.025
Diabetes-related medications, *n* (%)				
Metformin	98,254 (44.5)	73,888 (43.2)	24,366 (49.0)	0.115
Sulfonylurea	55,625 (25.2)	39,657 (23.2)	15,968 (32.1)	0.200
DPP-4 inhibitor	49,594 (22.5)	35,522 (20.8)	14,072 (28.3)	0.175
Meglitinide	4,590 (2.1)	3,287 (1.9)	1,303 (2.6)	0.047
Thiazolidinedione	9,452 (4.3)	7,305 (4.3)	2,147 (4.3)	0.002
α-glucosidase inhibitor	9,515 (4.3)	6,871 (4.0)	2,644 (5.3)	0.061
Insulin	59,253 (26.9)	36,318 (21.2)	22,935 (46.1)	0.545
GLP-1 receptor agonist	803 (0.4)	671 (0.4)	132 (0.3)	0.022
SGLT2 inhibitor	9,638 (4.4)	7,965 (4.7)	1,673 (3.4)	0.066
Hypertension-related medications, *n* (%)				
ARB	74,882 (33.9)	55,158 (32.3)	19,724 (39.6)	0.154
ACE inhibitor	16,439 (7.5)	11,842 (6.9)	4,597 (9.2)	0.085
Calcium channel blocker	78,849 (35.7)	55,070 (32.2)	23,779 (47.8)	0.322
Diuretics	56,322 (25.5)	36,661 (21.5)	19,661 (39.5)	0.400
Beta-blocker	51,647 (23.4)	38,099 (22.3)	13,548 (27.2)	0.115
Dyslipidemia-related medications, *n* (%)				
Statin	93,534 (42.4)	71,594 (41.9)	21,940 (44.1)	0.045
Ezetimibe	12,927 (5.9)	10,393 (6.1)	2,534 (5.1)	0.043
Fibrate	7,470 (3.4)	5,935 (3.5)	1,535 (3.1)	0.022
Antiplatelet agents, *n* (%)				
Aspirin	63,581 (28.8)	45,716 (26.7)	17,865 (35.9)	0.198
Clopidogrel	38,793 (17.6)	26,321 (15.4)	12,472 (25.1)	0.242
Cilostazol	15,703 (7.1)	11,231 (6.6)	4,472 (9.0)	0.090
Glycoprotein IIb/IIIa antagonist	1,095 (0.5)	701 (0.4)	394 (0.8)	0.049

ACE, angiotensin-converting enzyme; ALP, alkaline phosphatase; ALT, alanine aminotransferase; ARB, angiotensin II receptor blocker; AST, alanine aminotransferase; BP, blood pressure; DPP-4, dipeptidyl peptidase-4; ESRD, end-stage renal disease; GGT, gamma-glutamyl transferase; GLP-1, glucagon-like peptide-1; HbA1c, glycated hemoglobin; HDL, high-density lipoprotein; LDL, low-density lipoprotein; SD, standard deviation; SGLT2, sodium-glucose cotransporter 2.

After 8:2 train–test split, 176,576 and 44,144 patients were enrolled in the training and testing sets, respectively. We found that 22.5% of the patients visited the ED during the same period in both the training and test sets. Although statistical differences were present in certain comorbidities between the training and test sets owing to the large sample size, the proportions of patients diagnosed with hypertension and dyslipidemia were roughly similar, at approximately 68% and 51%, respectively (Fig 1).

### ED visit prediction model development

Several ML models were evaluated using the hold-out validation set to predict ED visits among patients with T2DM (Table 2). Based on internal validation results, the CatBoost model demonstrated the highest AUROC (0.869) and AUPRC (0.756), followed closely by LightGBM (AUROC 0.868, AUPRC 0.751) and XGBoost (AUROC 0.866, AUPRC 0.747). Among the gradient boosting-based models, CatBoost provided a well-balanced performance profile — sensitivity 72.5%, specificity 85.9%, PPV 59.9%, and NPV 91.5% — alongside its superior discrimination metrics. The Random Forest model achieved the highest sensitivity (79.4%) among all models but at the cost of the lowest specificity (64.1%) and PPV (39.2%), attributable to its lowest threshold value (0.118). In contrast, the Decision Tree model exhibited the highest specificity (88.5%) among all models but showed the lowest AUROC (0.703) and AUPRC (0.478), indicating poor overall discriminative ability. The Neural Network achieved the highest PPV (60.6%) among all models, while LightGBM demonstrated the highest NPV (91.7%). The Extra Trees model showed intermediate AUROC (0.804) and AUPRC (0.621), with moderate sensitivity (71.6%) and specificity (75.1%). Logistic regression achieved an AUROC of 0.731 and an AUPRC of 0.474, with an accuracy of 69.2%, sensitivity of 65.9%, specificity of 70.2%, PPV of 39.1%, and NPV of 87.6% at an optimal threshold of 0.217. Overall, gradient boosting–based models (CatBoost, LightGBM, and XGBoost) consistently outperformed tree-based models, neural networks, and the logistic regression baseline in terms of both AUROC and AUPRC (Table 2).

**Table 2:** Internal validation performance metrics of each AutoML model and conventional statistical model across optimal thresholds on the hold-out dataset.

Model	AUROC	AUPRC	Accuracy, %	Sensitivity, %	Specificity, %	PPV, %	NPV, %	Threshold^*^
AutoML models								
Decision Tree	0.703	0.478	79.8	49.9	88.5	55.9	85.9	0.392
XGBoost	0.866	0.747	82.7	71.7	85.9	59.7	91.2	0.251
Random Forest	0.786	0.593	67.5	79.4	64.1	39.2	91.4	0.118
LightGBM	0.868	0.751	81.3	74.1	83.4	56.5	91.7	0.228
CatBoost	0.869	0.756	82.9	72.5	85.9	59.9	91.5	0.246
Extra Trees	0.804	0.621	74.3	71.6	75.1	45.6	90.1	0.200
Neural Network	0.847	0.702	82.7	66.9	87.3	60.6	90.1	0.277
Conventional statistical model								
Logistic Regression	0.731	0.474	69.2	65.9	70.2	39.1	87.6	0.217

* Optimal threshold identified by Youden’s Index.

AUROC, area under the receiver operating characteristic curve; AUPRC, area under the precision-recall curve; PPV, Positive Predictive Value; NPV, Negative Predictive Value; AutoML, automated machine learning; LightGBM, light gradient boosting machine; XGBoost, extreme gradient boosting; CatBoost, categorical boosting; Extra Trees, extremely randomized trees.

Based on internal validation performance, the CatBoost model was selected as the final predictive model (Fig 2) and subsequently evaluated on an independent test set. On the test set, the CatBoost model achieved an AUROC of 0.866 (95% CI, 0.862–0.871). The model also demonstrated an accuracy of 87.0% (95% CI, 86.7–87.3), a sensitivity of 54.1% (95% CI, 53.1–55.0), and a specificity of 96.6% (95% CI, 96.4–96.8). To assess within-network generalizability, the final CatBoost model was additionally evaluated on each of the five participating institutions’ subsets within the internal hold-out test set; site-level performance metrics are summarized in S4 Table.

**Fig 3:**
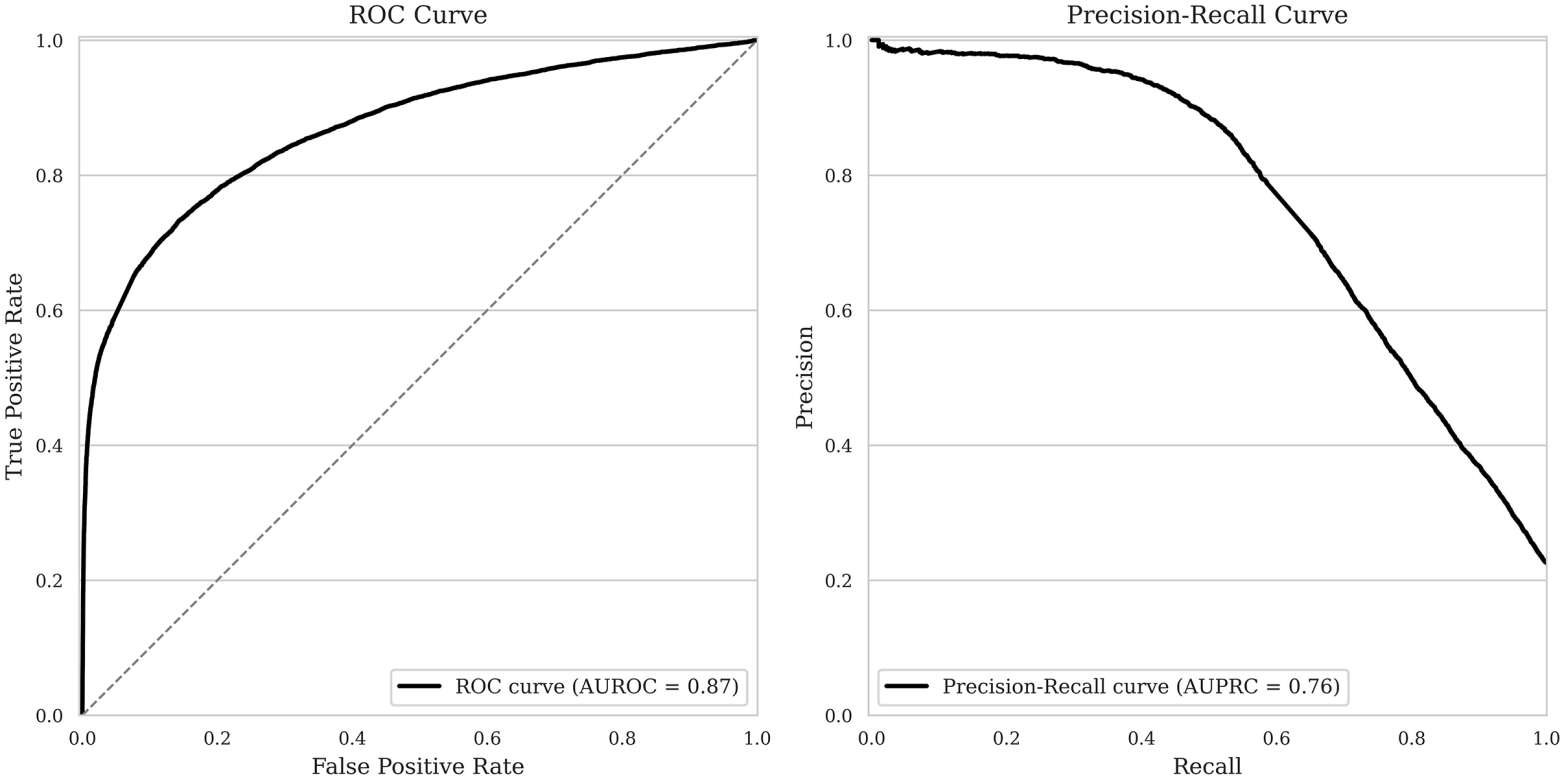
ROC curves of the CatBoost model. †ROC curve of the CatBoost model evaluated on the test set. ROC, receiver operating characteristic; AUROC, area under the receiver operating characteristic curve; AUPRC, area under the precision-recall curve.

The receiver operating characteristic (ROC) curve and precision–recall curve for the test set performance are presented in Fig 3. These findings indicate that the CatBoost model exhibits stable discriminative performance and may have potential utility in predicting ED visits among patients with T2DM.

Beyond discrimination, model calibration was evaluated on the internal hold-out test set. The final CatBoost model demonstrated excellent calibration, with a Brier score of 0.0998, well below the commonly cited threshold of 0.25 for clinically useful prediction and at the boundary of the 0.10 cut-off often used to define a well-calibrated model. The calibration slope was 1.03 and the calibration intercept was 0.04, both indicating that the predicted probabilities aligned almost perfectly with the observed event rates without any meaningful systematic over- or under-prediction. The reliability diagram (S1 Fig) further showed that the model’s predictions tracked the observed proportions closely across the full probability range, with all calibration deciles lying near the 45-degree line of perfect agreement [24,25]. Collectively, these results indicate that the proposed model is well-calibrated and can be applied directly without the need for post-hoc recalibration in populations comparable to the development cohort.

To examine the clinical utility of the model beyond conventional discrimination metrics, we conducted a DCA on the internal hold-out test set across threshold probabilities ranging from 0 to 0.5 (S2 Fig). The CatBoost model yielded a higher net benefit than both the “treat-all” and “treat-none” reference strategies across the entire range of clinically plausible decision thresholds, indicating that using the model to guide intervention decisions would result in a net gain in true positives without a disproportionate increase in unnecessary interventions. The advantage of the model over the “treat-all” strategy became more pronounced at higher threshold probabilities, where the relative cost of intervening in low-risk patients is greatest. These findings support the potential value of the proposed model as a risk-stratification tool for prioritizing closer monitoring or preventive interventions among patients with T2DM at elevated risk of emergency department utilization.

Taken together, these findings demonstrate that the CatBoost model provides meaningful improvements over a conventional statistical baseline and exhibits robust discriminative performance, well-calibrated risk predictions, and meaningful clinical utility for identifying patients with T2DM at elevated risk of ED visits.

### ED visit prediction performance in the final model

**Fig 4:**
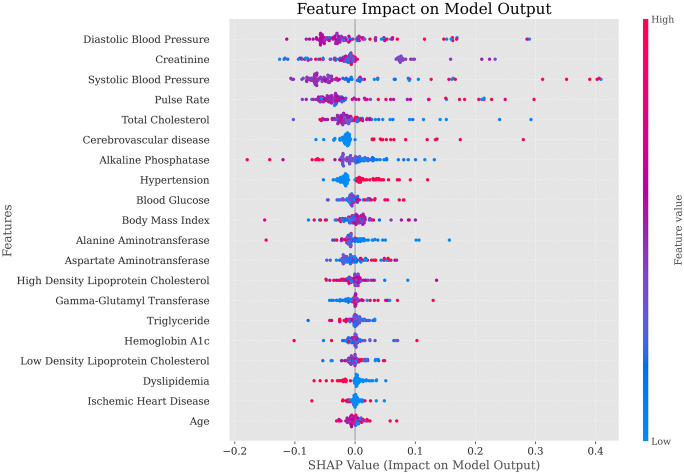
Top 20 feature importances of the CatBoost model.

SHAP (SHapley Additive exPlanations) values were used to demonstrate how the CatBoost model classified ED visits using the variables. We extracted 100 individual patient records randomly selected from the independent test set to interpret SHAP. Next, the mean absolute SHAP values were calculated to identify the key variables that most significantly impacted model predictions. SHAP serves as a tool for visualizing the importance of each variable, aiding the interpretation of the model’s prediction results. In the final model, the top 20 features with the highest predictive power among the 55 incorporated features were diastolic blood pressure (0.07), creatinine, systolic blood pressure, pulse rate, total cholesterol, cerebrovascular disease, ALP, hypertension, blood glucose, BMI, ALT, AST, HDL cholesterol, GGT, triglyceride, HbA1c, LDL cholesterol, dyslipidemia, ischemic heart disease, and age (Fig 4).

To assess the stability of SHAP-derived feature importance rankings across institutions, institution-specific SHAP analyses were additionally conducted by randomly selecting 100 patients from each institution’s subset of the hold-out test set and computing SHAP values independently for each institutional subset (S3 Fig). Six features — creatinine, diastolic blood pressure, systolic blood pressure, pulse rate, total cholesterol, and alkaline phosphatase — consistently ranked within the top 10 predictors across all five institutions. AST and blood glucose appeared in the top 10 in four of the five institutions. The broad pattern of importance, in which vital signs, renal function markers, lipid-related variables, and hepatic enzymes dominated the rankings, was consistently observed across all institutions, supporting the robustness of the feature importance structure across the heterogeneous multi-institutional study population.

## Discussion

In this multi-institutional study of 220,720 patients with T2DM, a CatBoost-based predictive model achieved an AUROC of 0.869 on internal validation and 0.866 (95% CI, 0.862–0.871) on the independent test set, demonstrating robust discriminative performance for identifying patients at risk of ED visits. SHAP values were applied to elucidate key predictors such as diastolic blood pressure and creatinine levels, offering a more nuanced understanding of the factors influencing ED visits in a large-scale, multi-institutional Korean cohort. The primary contribution of this work lies in the rigorous application and comparative evaluation of established ML approaches using real-world EHR data from five tertiary centers — a clinical context in which such systematic evaluation had not previously been conducted — thereby providing actionable and interpretable insights to support clinical decision-making in T2DM management.

ML approaches have demonstrated utility across various aspects of T2DM risk stratification, including the early detection and diagnosis of T2DM itself [28–32]. However, their application to predicting ED visits in patients with already-established T2DM — a clinically distinct and arguably more urgent prediction task — has remained comparatively underexplored. In this study, among the seven ML algorithms evaluated within an AutoML framework, CatBoost demonstrated the best overall discriminative performance, achieving the highest AUROC (0.869) and AUPRC (0.756), along with the highest accuracy (82.9%) among all evaluated models, suggesting its strength in handling complex, high-dimensional EHR data with nonlinear feature relationships. The overall performance profile of CatBoost on internal validation — particularly its favorable balance of sensitivity, specificity, and PPV — supports its clinical relevance for identifying high-risk patients while maintaining an acceptable false-positive rate. Furthermore, the integration of SHAP values represents a novel contribution to the field, facilitating the interpretation of model outputs and enabling healthcare providers to identify and prioritize interventions based on the most impactful predictors [33]. To our knowledge, this study represents one of the first large-scale, multi-institutional ML-based analyses of all-cause ED visit prediction in patients with T2DM in South Korea, extending prior work in both the breadth of clinical variables incorporated and the diversity of the study population.

Among studies specifically addressing ED or acute healthcare utilization in patients with T2DM, Karter et al. developed and externally validated an ML-based risk stratification tool to identify T2DM patients at high risk of hypoglycemia-related ED or hospital use over a 12-month horizon [34]. Using data from over 200,000 patients and validated across more than 1.3 million patients in independent cohorts, their tool — based on only six clinical variables — demonstrated good discrimination and broad transportability across healthcare systems. However, their model was specifically designed for hypoglycemia-related utilization and relied on a small number of predefined variables, potentially limiting its applicability to the full spectrum of diabetes-related ED presentations. In contrast, our model was developed to predict all-cause first ED visits in T2DM patients, incorporating a comprehensive set of 55 variables — including dynamic laboratory parameters, medication histories, and comorbidity profiles — across a large multi-institutional Korean cohort, thereby broadening both the clinical scope and the generalizability of the prediction framework.

More broadly, prior studies have attempted to predict the risk of ED visits in general populations, though fewer have focused specifically on patients with T2DM. For example, a US study utilized demographic and underlying disease data to forecast ED visits among community-dwelling older adults; however, it did not incorporate modifiable variables [35]. Similarly, although a Scottish study predicted emergency admissions based on medication usage in patients over 40 years of age [36], it failed to consider recently prescribed medications, complicating its applicability due to reliance on prescriptions from 3 years earlier. Another study employed primary care data to predict emergency hospitalizations but limited the adjustable variables to six drug classes [37].

In contrast, the present study offers several specific advantages over the prior models described above. First, whereas Crane et al. [35], Donnan et al. [36], and Hippisley-Cox & Coupland [37] developed general-population ED or emergency admission prediction models that were not tailored to T2DM, our model was designed exclusively for patients with established T2DM — a population with a distinct risk profile driven by disease-specific factors such as glycemic variability, medication complexity, and diabetes-related comorbidities. Second, our model incorporated a comprehensive set of 55 dynamic clinical variables — including serial laboratory values, detailed medication histories covering nine antidiabetic drug classes, and comorbidity profiles — compared to the more limited or static variable sets used in the preceding models. Third, the gradient boosting-based CatBoost algorithm, optimized via AutoML, achieved an AUROC of 0.866 on the independent test set, comparing favorably with the C-statistics reported for PEONY (0.79) and QAdmissions (0.76). Together, these features contribute to a more targeted and clinically actionable risk stratification tool for the T2DM population [38].

By integrating multi-institutional EHR data across five tertiary centers, this study captured a broad and clinically heterogeneous T2DM population, enhancing the representativeness of the derived risk model. The predictive model identifies high-risk patients by generating individualized risk scores based on routinely documented clinical variables — including laboratory values, vital signs, medication histories, and comorbidity profiles — that are already available in the EHR at the time of outpatient encounters. This allows the model to be applied prospectively at the point of care, without requiring additional testing or procedures, to flag patients whose clinical profiles are predictive of a future ED visit. Importantly, the intended deployment scenario is institution-specific rather than cross-institutional: the model would be integrated into each hospital’s local EMR system and applied at the time of scheduled outpatient visits, using clinical variables already documented in that encounter. This encounter-based framework circumvents the practical challenges of cross-institutional data synchronization and EMR update delays, as the model operates solely on data available within the local EHR environment at the moment of clinical decision-making. Clinicians can then act on this information through targeted preventive measures — such as medication optimization, intensified follow-up scheduling, or timely specialist referral — before acute decompensation occurs. SHAP-based interpretability further supports individualized decision-making by identifying which specific variables contributed most to each patient’s predicted risk, providing clinically actionable insights alongside the model output.

In patients with T2DM, failure to identify high-risk individuals in a timely manner can result in avoidable acute complications — including severe hypoglycemia, hyperglycemic crises, and cardiovascular events — that frequently culminate in ED visits. If prospectively validated and integrated into clinical workflows, the present model could support a shift from reactive to proactive management by prompting earlier, targeted outpatient interventions — such as medication adjustment, intensified monitoring, or timely specialist referral — before acute decompensation occurs. However, we acknowledge that actual clinical deployment would require prospective validation and implementation studies prior to routine use.

Beyond providing a binary risk classification, the SHAP-based interpretability of the model allows primary care providers to understand which specific clinical variables are driving an individual patient’s elevated risk — enabling genuinely personalized preventive action rather than a uniform protocol applied to all high-risk patients. For example, if a patient’s SHAP profile indicates that diastolic blood pressure and serum creatinine are the dominant contributors to their predicted risk, the clinician may prioritize antihypertensive regimen optimization and nephrology referral. In this way, the combination of a risk score and patient-level SHAP values provides a clinically actionable and individualized roadmap for preventive intervention, supporting targeted decision-making at the point of care.

This study highlights the potential of predictive models; however, we have also identified certain limitations and challenges that must be addressed for effective clinical application. First, the study relied on EMR for data collection; however, the lack of data quality and standardization undermined the model’s accuracy and reliability. Issues such as incomplete or biased data may distort predictions, necessitating additional efforts for data refinement and standardization. Second, although the model was trained and tested using data from five hospitals, it lacked validation using external datasets. Future research should test the model using data from other institutions or temporally distinct datasets to enhance generalizability and ensure robust performance across diverse settings. Third, the sensitivity of the model was approximately 54%, raising concerns regarding its ability to effectively identify high-risk patients. This limitation may have resulted in missed clinically important cases. However, the high specificity of the model reduces false positives, minimizes unnecessary alerts, and optimizes resource use, particularly in high-pressure environments such as EDs. Fourth, our feature engineering approach utilized summary statistics (median and standard deviation) of clinical parameters across the observation period to capture the chronic phenotypic vulnerability of patients with T2DM. While this mitigates short-term measurement noise, it introduces temporal overlap between the predictors and the index event. Therefore, our model functions primarily as a risk-stratification tool for identifying high-risk patient profiles rather than a prospective, real-time early warning system. Future studies aiming to develop real-time predictive models must ensure strict temporal separation by restricting predictors to data available exclusively prior to the index event. Fifth, sensitivity analyses comparing the Multivariate Imputation by Chained Equations (MICE)-based imputation approach against alternative strategies — such as complete-case analysis, mean or median imputation, or multiple imputation with Rubin’s rules pooling — were not performed in this study. Given the substantial proportion of missing data across several continuous predictors (ranging from 19.5% to 73.4%), the potential impact of the imputation strategy on model performance cannot be fully excluded, and future studies should evaluate the robustness of the findings to alternative missing data handling approaches. Finally, while the model is designed for encounter-based, single-instance inference within each institution’s local EMR environment — which mitigates concerns about real-time computational latency — broader clinical deployment would require careful attention to IT infrastructure integration, periodic model retraining as clinical practice patterns evolve, and ongoing model monitoring and maintenance, all of which represent recognized challenges in the sustainable implementation of ML-based clinical decision support tools [39–41].

Nevertheless, this study represents a significant milestone as the first application of ML-based prediction of ED visits among patients with T2DM using large-scale, multi-institutional EHR data from five tertiary medical centers across multiple regions of South Korea. Our findings suggest that implementing evidence-based individualized preventive interventions can substantially alleviate the burden of ED visits among Korean patients with diabetes.

This research represents an initial exploration of the potential of predictive modeling; future studies are required to validate the effectiveness and generalizability of the model. Evaluating the performance of the model across diverse healthcare settings and conducting external validation using independent datasets are crucial for ensuring its robustness. In addition, intervention studies are required to assess the model’s contribution to reducing the frequency of ED visits and improving patient health outcomes. Future research should develop user-friendly and intuitive interfaces and educational tools to help patients better understand their predicted risk of ED visits and utilize this information to improve diabetes management.

## Conclusion

This study validates the effectiveness of ML models in predicting ED visits among patients with T2DM using a large-scale, multi-center cohort. Beyond discriminative performance, SHAP-based interpretability provides clinically meaningful insights into the key modifiable risk factors driving individual predictions. If prospectively validated, this tool could support clinicians in prioritizing targeted preventive actions — such as medication regimen review, glycemic target reassessment, intensified outpatient monitoring, or timely specialist referral — tailored to each patient’s predicted risk level and the specific variables identified as most influential by the model. Ultimately, if integrated into primary care workflows, this approach may contribute to a shift from reactive to proactive management of T2DM, with the potential to reduce the burden of avoidable acute complications, though formal implementation and intervention studies will be necessary to confirm its real-world clinical impact.

## Supporting information

S1 Fig Calibration plot of the CatBoost model on the independent test set. The calibration plot displays the agreement between the mean predicted probability (x-axis) and the observed fraction of positive outcomes (y-axis) across deciles of predicted risk. The dotted diagonal line represents perfect calibration, in which predicted probabilities exactly correspond to observed event rates. The solid blue line with square markers represents the CatBoost model.
(TIF)

S2 Fig Decision curve analysis of the CatBoost model on the independent test set. The decision curve analysis displays the net benefit (y-axis) of the CatBoost model across a range of threshold probabilities from 0 to 0.5 (x-axis). The green line represents the CatBoost model, the red line represents the “treat all” strategy (i.e., assuming all patients will visit the ED), and the blue line represents the “treat none” strategy (i.e., assuming no patients will visit the ED). ED, emergency department.
(TIF)

S3 Fig Institution-specific SHAP (SHapley Additive exPlanations) feature importance plots for the final CatBoost model. Each panel displays the SHAP value distribution for the top 20 predictors derived from 100 patients randomly selected from each institution’s subset of the hold-out test set: AUMC, KHMC, KNUH, SJMC, and WKUH. AUMC, Ajou University Medical Center; KHMC, Kyung Hee Medical Center; KNUH, Kangwon National University Hospital; SJMC, Bucheon Sejong Hospital; WKUH, Wonkwang University Hospital; BP, blood pressure.
(TIF)

S1 Table Missingness of continuous predictor variables. (DOCX)

S2 Table Tuned hyperparameters of the final CatBoost model selected by the MLJAR-supervised AutoML framework. (DOCX)

S3 Table Baseline characteristics of the study cohort stratified by participating hospital site. (DOCX)

S4 Table Performance metrics of the final CatBoost model stratified by participating hospital site. (DOCX)
