# Supplementary figures and images for: Machine learning for predicting emergency department visits in patients with type 2 diabetes: A real-world, multi-institutional study
Source: PLoS One. 2026 Jul 9;21(7):e0352342. doi: 10.1371/journal.pone.0352342 (PMC13349136; doi:10.1371/journal.pone.0352342)

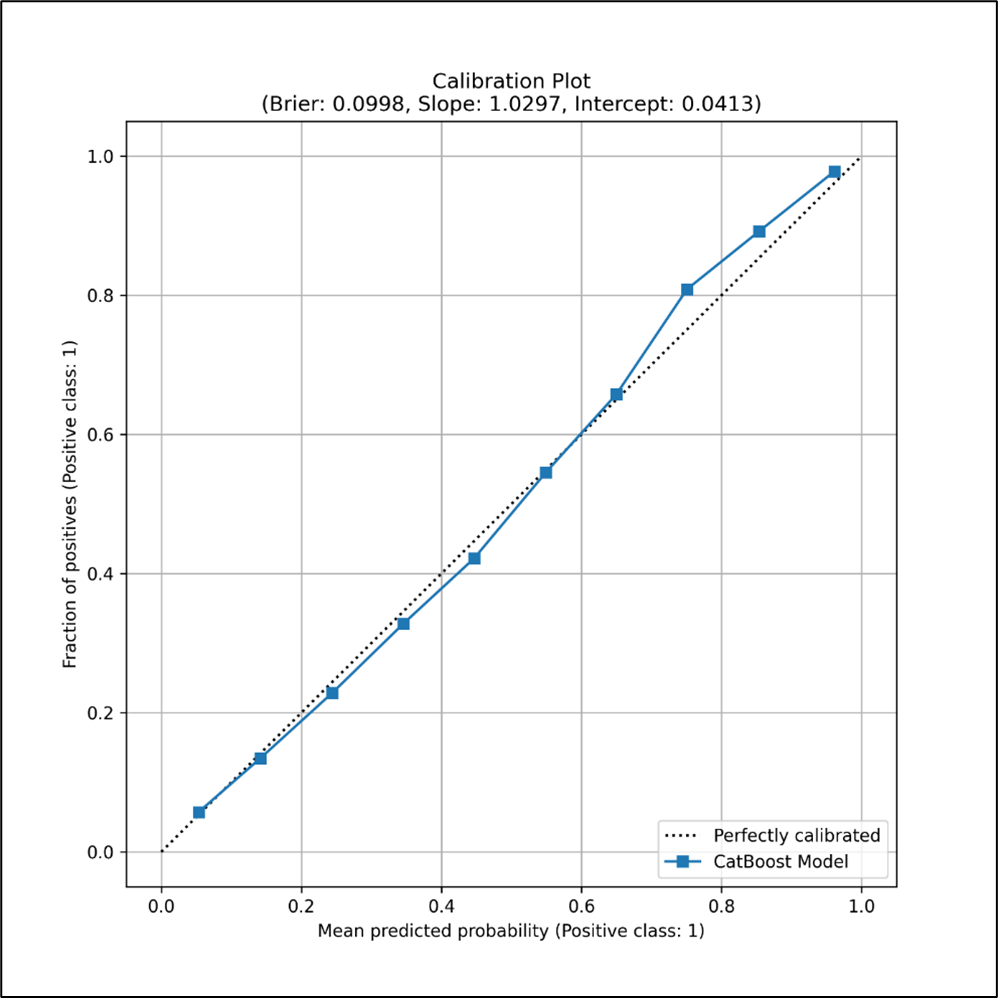

Supplement: S1 Fig — The calibration plot displays the agreement between the mean predicted probability (x-axis) and the observed fraction of positive outcomes (y-axis) across deciles of predicted risk. The dotted diagonal line represents perfect calibration, in which predicted probabilities exactly correspond to observed event rates. The solid blue line with square markers represents the CatBoost model. (TIF) [file pone.0352342.s001.tif]

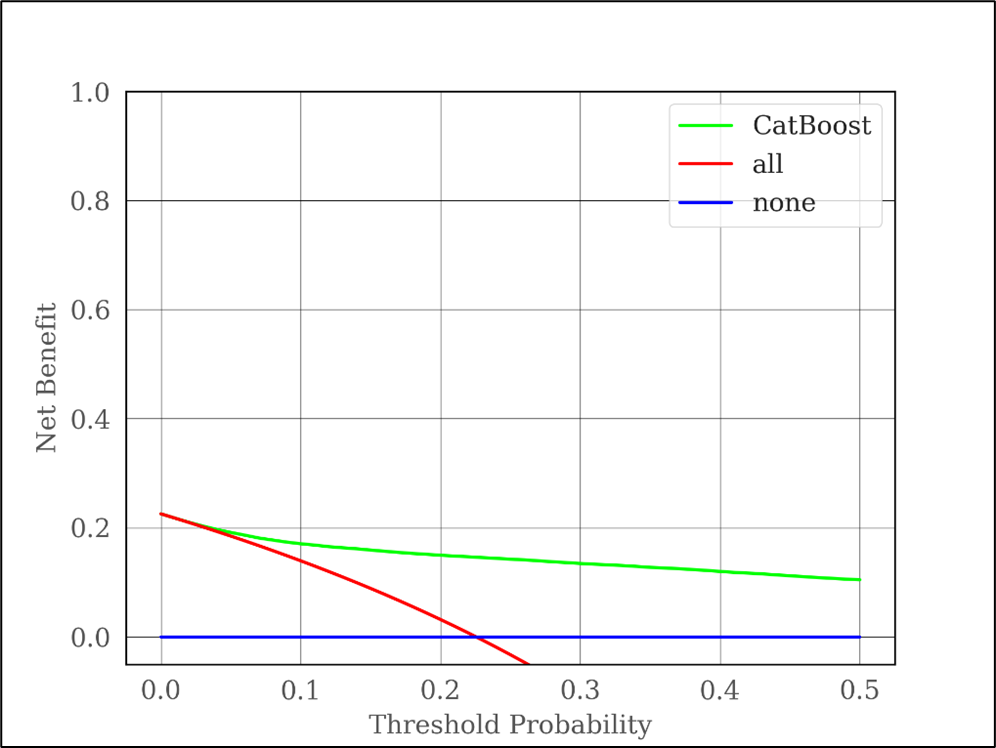

Supplement: S2 Fig — The decision curve analysis displays the net benefit (y-axis) of the CatBoost model across a range of threshold probabilities from 0 to 0.5 (x-axis). The green line represents the CatBoost model, the red line represents the “treat all” strategy (i.e., assuming all patients will visit the ED), and the blue line represents the “treat none” strategy (i.e., assuming no patients will visit the ED). ED, emergency department. (TIF) [file pone.0352342.s002.tif]

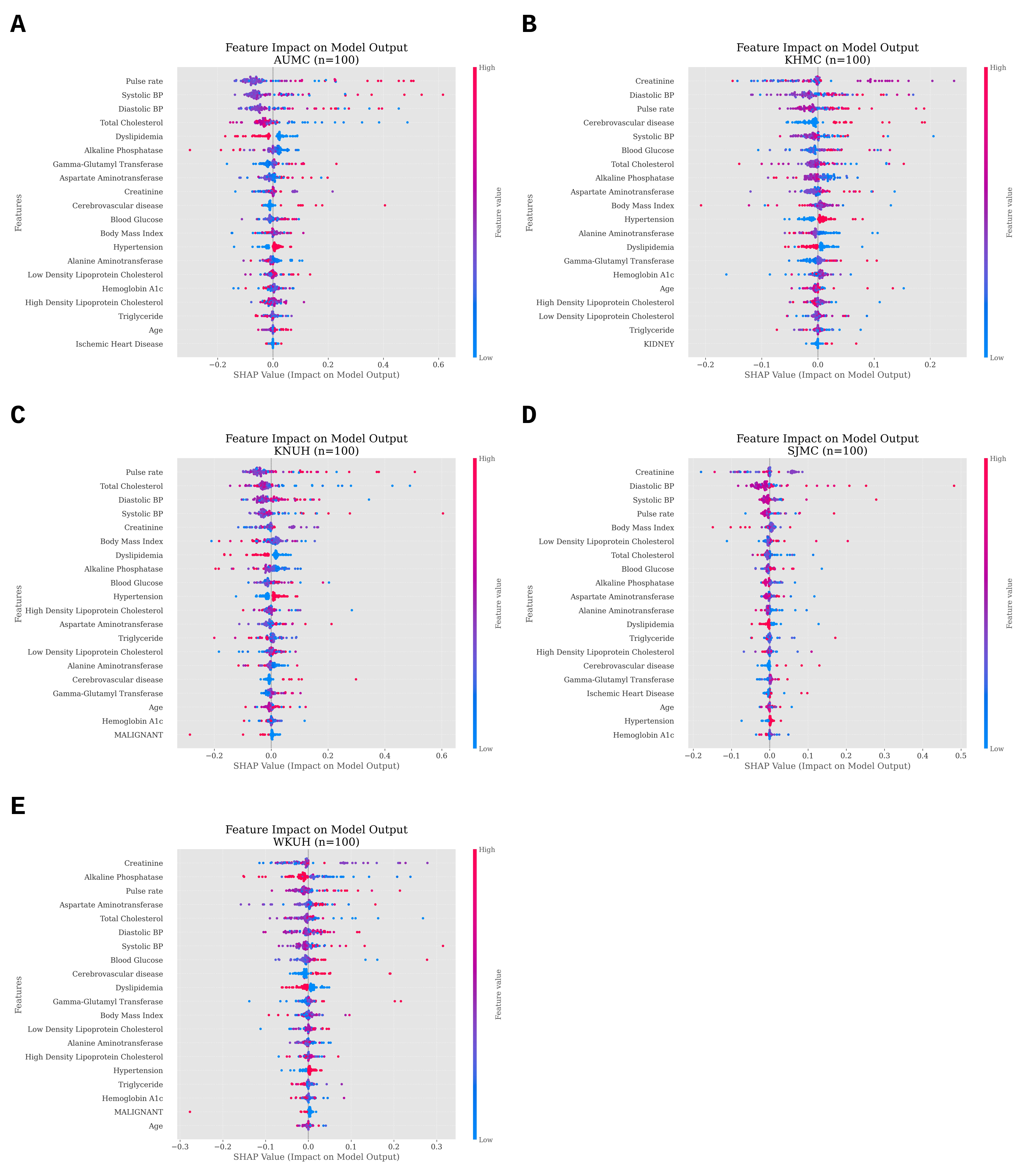

Supplement: S3 Fig — Each panel displays the SHAP value distribution for the top 20 predictors derived from 100 patients randomly selected from each institution’s subset of the hold-out test set: AUMC, KHMC, KNUH, SJMC, and WKUH. AUMC, Ajou University Medical Center; KHMC, Kyung Hee Medical Center; KNUH, Kangwon National University Hospital; SJMC, Bucheon Sejong Hospital; WKUH, Wonkwang University Hospital; BP, blood pressure. (TIF) [file pone.0352342.s003.tif]
