# Supplementary material for: Machine learning for predicting emergency department visits in patients with type 2 diabetes: A real-world, multi-institutional study
Source: PLoS One. 2026 Jul 9;21(7):e0352342. doi: 10.1371/journal.pone.0352342 (PMC13349136; doi:10.1371/journal.pone.0352342)
Supplement: S1 Table — (DOCX) [file pone.0352342.s004.docx]

**S1 Table.** Missingness of continuous predictor variables.

| Variable (full term) | Abbreviation | Unit | N observed | N missing | Missing (%) |
| --- | --- | --- | --- | --- | --- |
| Body Mass Index | BMI | kg/m² | 58,631 | 162,089 | 73.44 |
| Gamma-Glutamyl Transferase | GGT | U/L | 86,348 | 134,372 | 60.88 |
| Pulse Rate | PR | beats/min | 99,943 | 120,777 | 54.72 |
| Systolic Blood Pressure | SBP | mmHg | 104,289 | 116,431 | 52.75 |
| Diastolic Blood Pressure | DBP | mmHg | 104,806 | 115,914 | 52.52 |
| Low-Density Lipoprotein Cholesterol | LDL-C | mg/dL | 106,042 | 114,678 | 51.96 |
| High-Density Lipoprotein Cholesterol | HDL-C | mg/dL | 114,962 | 105,758 | 47.92 |
| Triglyceride | TG | mg/dL | 120,963 | 99,757 | 45.20 |
| Hemoglobin A1c | HbA1c | % | 147,697 | 73,023 | 33.08 |
| Total Cholesterol | TC | mg/dL | 154,526 | 66,194 | 29.99 |
| Alkaline Phosphatase | ALP | U/L | 164,029 | 56,691 | 25.68 |
| Blood Glucose | Glucose | mg/dL | 165,818 | 54,902 | 24.87 |
| Aspartate Aminotransferase | AST | U/L | 176,449 | 44,271 | 20.06 |
| Alanine Aminotransferase | ALT | U/L | 176,587 | 44,133 | 20.00 |
| Creatinine | Cr | mg/dL | 177,604 | 43,116 | 19.53 |
| All 15 variables (mean) |  |  | — | — | 40.84 |
| Patients with complete data on all 15 variables |  |  | 16,572 (7.51%) | 204,148 (92.49%) | — |
