# Supplementary material for: Machine learning for predicting emergency department visits in patients with type 2 diabetes: A real-world, multi-institutional study
Source: PLoS One. 2026 Jul 9;21(7):e0352342. doi: 10.1371/journal.pone.0352342 (PMC13349136; doi:10.1371/journal.pone.0352342)
Supplement: S2 Table — (DOCX) [file pone.0352342.s005.docx]

**S2 Table.** Tuned hyperparameters of the final CatBoost model selected by the MLJAR-supervised AutoML framework.

| Hyperparameter | Value |
| --- | --- |
| learning_rate | 0.1 |
| depth | 6 |
| rsm (feature subsample) | 1.0 |
| l2_leaf_reg | 3 (default) |
| border_count | 254 (default) |
| bootstrap_type | Bayesian (default) |
| grow_policy | SymmetricTree |
| leaf_estimation_method | Newton |
| loss_function | Logloss |
| eval_metric | AUC |
| iterations (max) | 10,000 |
| early_stopping_rounds | 50 |
| Selected trees (best iteration) | 1,275 |
| random_seed | 6 |
| automl_random_state | 1234 |

Hyperparameters were identified through a random search (10 models per algorithm) followed by hill-climbing refinement (2 steps on the top 3 candidates), conducted within the MLJAR-supervised AutoML framework (mode = “Compete”; eval_metric = “auc”; total_time_limit = 3,600 s; random_state = 1234).

AUC, area under the receiver operating characteristic curve; AutoML, automated machine learning.
