# Supplementary material for: Machine learning for predicting emergency department visits in patients with type 2 diabetes: A real-world, multi-institutional study
Source: PLoS One. 2026 Jul 9;21(7):e0352342. doi: 10.1371/journal.pone.0352342 (PMC13349136; doi:10.1371/journal.pone.0352342)
Supplement: S3 Table — (DOCX) [file pone.0352342.s006.docx]

**S3 Table. Baseline characteristics of the study cohort stratified by participating hospital site.**

|  | **Overall**  **(220,720)** | **AUMC (68,932)** | **KHMC (64,436)** | **KNUH (17,894)** | **SJMC (38,195)** | **WKUH (31,263)** | **P-value** |
| --- | --- | --- | --- | --- | --- | --- | --- |
| ED visit, n (% within group) | 49,770 (22.5) | 29,718 (43.1) | 11,549 (17.9) | 3,411 (19.1) | 3,167 (8.3) | 1,925 (6.2) | <0.001 |
| Age, year, mean (SD) | 62.2 (13.8) | 60.7 (14.4) | 61.9 (13.9) | 63.1 (14.0) | 63.4 (12.1) | 64.0 (13.6) | <0.001 |
| Male, *n* (%) | 119,176 (54.0) | 39,501 (57.3) | 32,619 (50.6) | 9,822 (54.9) | 19,940 (52.2) | 17,294 (55.3) | <0.001 |
| BMI, kg/m^2^, mean (SD) | 25.1 (2.5) | 25.2 (2.0) | 25.0 (2.6) | 25.3 (3.0) | 25.2 (2.5) | 24.6 (2.6) | <0.001 |
| Systolic BP, mmHg, mean (SD) | 126.3 (16.7) | 126.0 (23.0) | 126.4 (12.0) | 130.1 (14.9) | 124.5 (10.8) | 126.6 (14.9) | <0.001 |
| Diastolic BP, mmHg, mean (SD) | 74.8 (9.9) | 74.6 (13.0) | 75.6 (7.7) | 76.6 (9.3) | 73.8 (6.9) | 73.9 (9.6) | <0.001 |
| Pulse rate, bpm, mean (SD) | 78.8 (11.7) | 79.5 (15.3) | 78.1 (9.3) | 82.4 (11.5) | 76.3 (7.8) | 79.3 (10.5) | <0.001 |
| Blood test**,** mean (SD) |  |  |  |  |  |  |  |
| Glucose, mg/dL | 149.6 (47.9) | 149.6 (51.3) | 146.8 (45.5) | 159.6 (53.8) | 144.8 (39.6) | 156.0 (49.1) | <0.001 |
| Total cholesterol, mg/dL | 164.5 (37.0) | 162.5 (41.6) | 168.4 (34.3) | 166.1 (38.7) | 159.8 (34.0) | 166.1 (32.9) | <0.001 |
| HDL cholesterol, mg/dL | 46.8 (10.0) | 46.2 (9.9) | 48.1 (10.0) | 45.0 (10.4) | 48.2 (10.4) | 45.1 (9.4) | <0.001 |
| LDL cholesterol, mg/dL | 93.2 (29.7) | 90.8 (32.2) | 97.1 (27.4) | 94.6 (31.1) | 90.2 (29.4) | 93.7 (26.6) | <0.001 |
| Serum creatinine, mg/dL | 0.9 (0.2) | 1.0 (0.3) | 0.8 (0.2) | 0.9 (0.2) | 0.9 (0.2) | 0.9 (0.3) | <0.001 |
| ALP, U/L | 87.8 (33.5) | 81.6 (29.2) | 81.1 (23.8) | 82.0 (26.6) | 78.0 (21.0) | 130.9 (42.1) | <0.001 |
| AST, U/L | 26.3 (9.3) | 26.2 (10.5) | 26.4 (8.4) | 27.8 (8.9) | 24.8 (7.8) | 27.1 (10.1) | <0.001 |
| ALT, U/L | 25.9 (13.1) | 26.2 (14.4) | 25.5 (12.2) | 27.5 (13.0) | 25.0 (11.5) | 26.0 (13.8) | <0.001 |
| GGT, U/L | 43.3 (26.3) | 45.1 (26.8) | 41.0 (25.8) | 44.9 (26.7) | 41.0 (20.7) | 46.0 (31.0) | <0.001 |
| HbA1c, % | 7.0 (1.1) | 7.2 (1.1) | 6.8 (1.1) | 7.3 (1.1) | 6.9 (1.0) | 7.3 (1.2) | <0.001 |
| Triglyceride, mg/dL | 146.7 (61.0) | 147.4 (60.4) | 146.6 (61.7) | 156.0 (66.7) | 143.5 (58.8) | 144.1 (59.5) | <0.001 |
| Co-morbid conditions, *n* (%) |  |  |  |  |  |  |  |
| Hypertension | 151,314 (68.6) | 46,422 (67.3) | 41,403 (64.3) | 10,354 (57.9) | 30,968 (81.1) | 22,167 (70.9) | <0.001 |
| Dyslipidemia | 112,486 (51.0) | 31,802 (46.1) | 32,250 (50.0) | 8,213 (45.9) | 25,805 (67.6) | 14,416 (46.1) | <0.001 |
| Macrovascular complications, *n* (%) |  |  |  |  |  |  |  |
| Ischemic heart disease | 38,314 (17.4) | 8,978 (13.0) | 7,913 (12.3) | 2,342 (13.1) | 14,681 (38.4) | 4,400 (14.1) | <0.001 |
| Myocardial infarction | 1,419 (0.6) | 371 (0.5) | 231 (0.4) | 87 (0.5) | 601 (1.6) | 129 (0.4) | <0.001 |
| Heart failure | 10,916 (4.9) | 2,469 (3.6) | 1,959 (3.0) | 771 (4.3) | 4,560 (11.9) | 1,157 (3.7) | <0.001 |
| Atrial fibrillation | 10,833 (4.9) | 2,238 (3.2) | 2,179 (3.4) | 691 (3.9) | 4,108 (10.8) | 1,617 (5.2) | <0.001 |
| Parkinson's disease | 1,377 (0.6) | 501 (0.7) | 337 (0.5) | 92 (0.5) | 133 (0.3) | 314 (1.0) | <0.001 |
| Dementia | 2,314 (1.0) | 305 (0.4) | 174 (0.3) | 344 (1.9) | 867 (2.3) | 624 (2.0) | <0.001 |
| Cerebrovascular disease | 26,882 (12.2) | 6,173 (9.0) | 11,456 (17.8) | 1,729 (9.7) | 2,068 (5.4) | 5,456 (17.5) | <0.001 |
| Peripheral vascular disease | 483 (0.2) | 243 (0.4) | 0 (0.0) | 45 (0.3) | 124 (0.3) | 71 (0.2) | <0.001 |
| Lower limb amputation | 600 (0.3) | 388 (0.6) | 14 (0.0) | 55 (0.3) | 21 (0.1) | 122 (0.4) | <0.001 |
| Microvascular complications, *n* (%) |  |  |  |  |  |  |  |
| Retinopathy | 9,649 (4.4) | 2,866 (4.2) | 2,423 (3.8) | 1,929 (10.8) | 78 (0.2) | 2,353 (7.5) | <0.001 |
| Proliferative diabetic retinopathy | 1,810 (0.8) | 735 (1.1) | 0 (0.0) | 179 (1.0) | 0 (0.0) | 896 (2.9) | <0.001 |
| Chronic kidney disease | 15,687 (7.1) | 6,077 (8.8) | 3,446 (5.3) | 1,004 (5.6) | 1,988 (5.2) | 3,172 (10.1) | <0.001 |
| ESRD | 846 (0.4) | 327 (0.5) | 0 (0.0) | 71 (0.4) | 155 (0.4) | 293 (0.9) | <0.001 |
| Diabetic neuropathy | 21,431 (9.7) | 4,262 (6.2) | 10,160 (15.8) | 1,657 (9.3) | 2,947 (7.7) | 2,405 (7.7) | <0.001 |
| Cancer, *n* (%) | 8,947 (4.1) | 16 (0.0) | 4,749 (7.4) | 946 (5.3) | 303 (0.8) | 2,933 (9.4) | <0.001 |
| Diabetes-related medications, *n* (%) |  |  |  |  |  |  |  |
| Metformin | 98,254 (44.5) | 32,159 (46.7) | 26,087 (40.5) | 10,152 (56.7) | 15,795 (41.4) | 14,061 (45.0) | <0.001 |
| Sulfonylurea | 55,625 (25.2) | 18,706 (27.1) | 14,497 (22.5) | 5,030 (28.1) | 9,062 (23.7) | 8,330 (26.6) | <0.001 |
| DPP-4 inhibitor | 49,594 (22.5) | 18,242 (26.5) | 11,485 (17.8) | 5,343 (29.9) | 7,120 (18.6) | 7,404 (23.7) | <0.001 |
| Meglitinide | 4,590 (2.1) | 898 (1.3) | 1,819 (2.8) | 81 (0.5) | 1,064 (2.8) | 728 (2.3) | <0.001 |
| Thiazolidinedione | 9,452 (4.3) | 2,875 (4.2) | 2,881 (4.5) | 575 (3.2) | 1,533 (4.0) | 1,588 (5.1) | <0.001 |
| α-glucosidase inhibitor | 9,515 (4.3) | 2,672 (3.9) | 1,892 (2.9) | 429 (2.4) | 2,800 (7.3) | 1,722 (5.5) | <0.001 |
| Insulin | 59,253 (26.8) | 22,611 (32.8) | 14,824 (23.0) | 4,108 (23.0) | 5,902 (15.5) | 11,808 (37.8) | <0.001 |
| GLP-1 receptor agonist | 803 (0.4) | 396 (0.6) | 183 (0.3) | 67 (0.4) | 46 (0.1) | 111 (0.4) | <0.001 |
| SGLT2 inhibitor | 9,638 (4.4) | 2,602 (3.8) | 1,561 (2.4) | 1,844 (10.3) | 2,563 (6.7) | 1,068 (3.4) | <0.001 |
| Hypertension-related medications, *n* (%) |  |  |  |  |  |  |  |
| ARB | 74,882 (33.9) | 23,071 (33.5) | 21,900 (34.0) | 5,583 (31.2) | 14,033 (36.7) | 10,295 (32.9) | <0.001 |
| ACE inhibitor | 16,439 (7.4) | 4,140 (6.0) | 2,931 (4.5) | 620 (3.5) | 6,075 (15.9) | 2,673 (8.6) | <0.001 |
| Calcium channel blocker | 78,849 (35.7) | 25,820 (37.5) | 24,327 (37.8) | 4,519 (25.3) | 13,027 (34.1) | 11,156 (35.7) | <0.001 |
| Diuretics | 56,322 (25.5) | 19,096 (27.7) | 14,520 (22.5) | 1,932 (10.8) | 11,339 (29.7) | 9,435 (30.2) | <0.001 |
| Beta-blocker | 51,647 (23.4) | 12,781 (18.5) | 11,687 (18.1) | 3,343 (18.7) | 17,450 (45.7) | 6,386 (20.4) | <0.001 |
| Dyslipidemia-related medications, *n* (%) |  |  |  |  |  |  |  |
| Statin | 93,534 (42.4) | 26,296 (38.1) | 27,067 (42.0) | 6,318 (35.3) | 21,667 (56.7) | 12,186 (39.0) | <0.001 |
| Ezetimibe | 12,927 (5.9) | 3,928 (5.7) | 3,394 (5.3) | 286 (1.6) | 3,432 (9.0) | 1,887 (6.0) | <0.001 |
| Fibrate | 7,470 (3.4) | 2,895 (4.2) | 1,905 (3.0) | 208 (1.2) | 1,411 (3.7) | 1,051 (3.4) | <0.001 |
| Antiplatelet agents, *n* (%) |  |  |  |  |  |  |  |
| Aspirin | 63,581 (28.8) | 16,502 (23.9) | 16,176 (25.1) | 4,954 (27.7) | 17,571 (46.0) | 8,378 (26.8) | <0.001 |
| Clopidogrel | 38,793 (17.6) | 10,846 (15.7) | 11,134 (17.3) | 2,161 (12.1) | 9,195 (24.1) | 5,457 (17.5) | <0.001 |
| Cilostazol | 15,703 (7.1) | 3,704 (5.4) | 4,803 (7.5) | 817 (4.6) | 3,018 (7.9) | 3,361 (10.8) | <0.001 |
| Glycoprotein IIb/IIIa antagonist | 1,095 (0.5) | 153 (0.2) | 350 (0.5) | 62 (0.3) | 304 (0.8) | 226 (0.7) | <0.001 |

ED visit, n (% within group) denotes the number of patients experiencing a diabetes-related ED visit within one year of the index visit (the primary outcome) and its prevalence within each group. Continuous variables are presented as mean (standard deviation) and categorical variables as number (percentage). P-values were derived from Kruskal-Wallis tests for continuous variables and Pearson's chi-square tests for categorical variables, comparing the five hospital sites (the Overall column was not included in the comparisons).

Given the very large sample size (n = 220,720), even small absolute differences across sites may reach statistical significance; p-values are therefore presented for descriptive purposes only and should be interpreted alongside the magnitude of the differences. P-values were not adjusted for multiple comparisons.

AUMC, Ajou University Medical Center; KHMC, Kyung Hee Medical Center; KNUH, Kangwon National University Hospital; SJMC, Bucheon Sejong Hospital; WKUH, Wonkwang University Hospital; SD, standard deviation; BMI, body mass index; BP, blood pressure; HDL, high-density lipoprotein; LDL, low-density lipoprotein; ALP, alkaline phosphatase; AST, aspartate aminotransferase; ALT, alanine aminotransferase; GGT, gamma-glutamyl transferase; HbA1c, glycated hemoglobin; ESRD, end-stage renal disease; ARB, angiotensin II receptor blocker; ACE, angiotensin-converting enzyme; DPP-4, dipeptidyl peptidase-4; GLP-1, glucagon-like peptide-1; SGLT2, sodium-glucose cotransporter-2; ED, emergency department.
