# Supplementary material for: Machine learning for predicting emergency department visits in patients with type 2 diabetes: A real-world, multi-institutional study
Source: PLoS One. 2026 Jul 9;21(7):e0352342. doi: 10.1371/journal.pone.0352342 (PMC13349136; doi:10.1371/journal.pone.0352342)
Supplement: S4 Table — (DOCX) [file pone.0352342.s007.docx]

**S4 Table. Performance metrics of the final CatBoost model stratified by participating hospital site.**

| **Site** | **AUROC** | **AUPRC** | **AUPRC Lift** | **Sensitivity, %** | **Specificity, %** | **PPV, %** | **NPV, %** | **Threshold^*^** |
| --- | --- | --- | --- | --- | --- | --- | --- | --- |
| AUMC | 0.975 | 0.971 | 2.267 | 92.5% | 95.3% | 93.7% | 94.4% | 0.212 |
| KHMC | 0.805 | 0.578 | 3.150 | 66.3% | 80.7% | 43.5% | 91.4% | 0.196 |
| KNUH | 0.719 | 0.416 | 2.190 | 73.3% | 59.5% | 29.8% | 90.5% | 0.138 |
| SJMC | 0.790 | 0.292 | 3.613 | 74.3% | 69.3% | 17.5% | 96.9% | 0.094 |
| WKUH | 0.697 | 0.160 | 2.484 | 60.9% | 67.0% | 11.3% | 96.1% | 0.140 |

Performance was evaluated on the internal hold-out test set (n=44,144; 20% of the pooled cohort), with each row reporting metrics computed independently on the subset of patients from each participating hospital. AUPRC Lift adjusts for the across-site variation in event prevalence, enabling fair cross-site comparison.

^*^ Optimal threshold identified independently for each site using Youden’s Index.

AUROC, area under the receiver operating characteristic curve; AUPRC, area under the precision-recall curve; AUPRC Lift, ratio of model AUPRC to random baseline (= prevalence); PPV, positive predictive value; NPV, negative predictive value; AUMC, Ajou University Medical Center; KHMC, Kyung Hee Medical Center; KNUH, Kangwon National University Hospital; SJMC, Bucheon Sejong Hospital; WKUH, Wonkwang University Hospital.
